# The Dual Role of A20 (TNFAIP3) in Viral Infection: A Context-Dependent Regulator of Immunity and Pathogenesis

**DOI:** 10.3390/v17121634

**Published:** 2025-12-17

**Authors:** Haesung Jeon, Choongho Lee

**Affiliations:** College of Pharmacy, Dongguk University-Seoul, Goyang 10326, Republic of Korea; hsj571148@dgu.ac.kr

**Keywords:** A20, NF-κB signaling, innate antiviral immunity, proviral–antiviral duality

## Abstract

A20 (TNFAIP3) is a ubiquitin-editing enzyme that plays a central role in the regulation of inflammation and cell death, primarily through modulation of NF-κB signaling. In the context of viral infection, A20 exhibits a dual nature: it can both suppress antiviral immune responses to facilitate viral replication and act as a host-protective factor to prevent immunopathology. This review synthesizes current findings on the context-dependent roles of A20, focusing on its capacity to switch between antiviral and proviral functions. We examine how specific determinants—including viral genetic makeup, the infected cell type, and the temporal stage of infection—dictate whether A20 protects the host or facilitates viral persistence. We propose a systematic framework for understanding A20 as a dynamic regulator that orchestrates the balance between pathogen clearance and tissue protection.

## 1. Introduction

Viral infections pose a major threat to human health, forcing host cells to rapidly sense invading pathogens and mount effective immune defenses. The innate immune system serves as the first line of protection, relying on pattern recognition receptors (PRRs) to detect viral nucleic acids such as double-stranded RNA and unmethylated CpG DNA [1,2]. Activation of these receptors initiates signaling cascades that induce type I interferons (IFNs), pro-inflammatory cytokines, and chemokines, establishing an antiviral state and recruiting immune cells [1,2].

Central to this response is the transcription factor nuclear factor-kappa B (NF-κB), a master regulator of genes involved in inflammation, immunity, and cell survival [3,4]. NF-κB activation ensures the production of cytokines, chemokines, and anti-apoptotic factors essential for controlling infection [3,4]. However, uncontrolled NF-κB signaling can result in chronic inflammation, autoimmune disease, and even cancer [5,6,7]. To prevent such immunopathology, its activity must be tightly regulated. A key negative feedback regulator is the ubiquitin-editing enzyme A20 (tumor necrosis factor alpha-induced protein 3, TNFAIP3), which balances effective antiviral responses with protection against tissue damage [1,2,3,4,8,9].

While A20 is broadly characterized as an anti-inflammatory and host-protective factor, assigning it a static label of “antiviral” or “proviral” fails to capture its dynamic nature. A growing body of evidence reveals that A20 functions as both a facilitator and inhibitor of viral infection, with outcomes that are profoundly dependent on biological context. Here, we define such context-dependence not merely as variability, but as a systematic functional switch wherein A20 aligns either with host defense or viral survival. This duality is governed by three principal biological determinants: the genetic makeup of the invading virus, the specific tissue or cell type infected, and the temporal stage of the immune response.

To provide a systematic framework for analyzing these varied roles, we classify A20’s interactions with viruses into four distinct categories. Proviral interactions occur when A20’s immunosuppressive functions are co-opted by the virus to promote its own replication and persistence. Antiviral interactions describe situations in which A20 acts to limit viral replication or prevent host pathogenesis, consistent with its canonical protective role. Dual interactions represent cases where the role of A20 shifts dynamically between antiviral and proviral states depending on the temporal or cellular context of infection. Finally, we address context-dependent effects, wherein A20’s influence extends beyond the direct virus–host conflict to affect systemic homeostasis, tissue integrity, and long-term immunological outcomes, as exemplified by chronic Human Immunodeficiency Virus (HIV) infection.

## 2. Molecular Mechanisms of A20 Regulation

### 2.1. Transcriptional Control of A20 Expression

**Figure 1:**
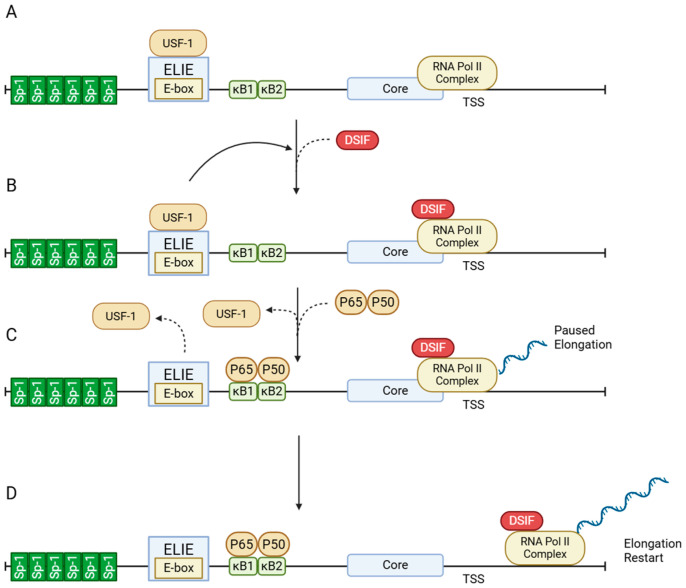
Structure and activation of the A20 promoter. (**A**) Promoter architecture. Upstream of the core promoter, A20 harbors two NF-κB sites (κB1/κB2), six Sp1 sites, and an E-box overlapping the Elongation-Inhibitory Element (ELIE) element. (**B**) Basal state. RNA polymerase II and the general transcription factors are bound at the core promoter, while the ELIE–USF1 axis suppresses transcript elongation through DRB Sensitivity-Inducing Factor (DSIF). The Sp1 cluster supports promoter accessibility and initiation capacity, but productive elongation is limited by DSIF. (**C**) Upon stimulation. NF-κB binds κB1/κB2 and displaces USF1; control of DSIF shifts to the NF-κB/core-promoter module, which triggers pause release and enables multiple rounds of transcription. (**D**) Elongation restart. DSIF converts to a pro-elongation role, allowing RNA polymerase II to transition from promoter-proximal pausing to productive mRNA elongation. Solid curved arrows indicate regulatory interactions, while dashed curved arrows represent the displacement or binding of transcription factors. Figure created with BioRender.com (https://BioRender.com/96ii9dl, accessed on 12 December 2025).

The control of A20 expression is achieved through transcriptional mechanisms that respond to viral stimuli. Figure 1 illustrates the architecture and activation of the A20 promoter. At baseline, A20 expression is tightly restricted by the interplay of transcription factors Sp1 and upstream stimulatory factor 1 (USF-1), which maintain RNA polymerase II in a paused state. Upon immune stimulation, NF-κB binds to κB sites, displaces USF-1, and facilitates transcriptional elongation, enabling prompt A20 induction. This inducible promoter design allows A20 to function as a negative feedback regulator, becoming upregulated when NF-κB activity peaks and establishing a self-limiting loop in inflammatory signaling [3].

### 2.2. Structural Organization and Catalytic Mechanisms

Structurally, A20 contains an N-terminal ovarian tumor (OTU) domain with deubiquitinase (DUB) activity and seven C-terminal zinc finger motifs, an arrangement that enables it to carry out two opposing enzymatic reactions simultaneously [3,4]. The structural organization of A20 and its regulatory complex provides the molecular foundation for its functional versatility.

**Figure 2:**
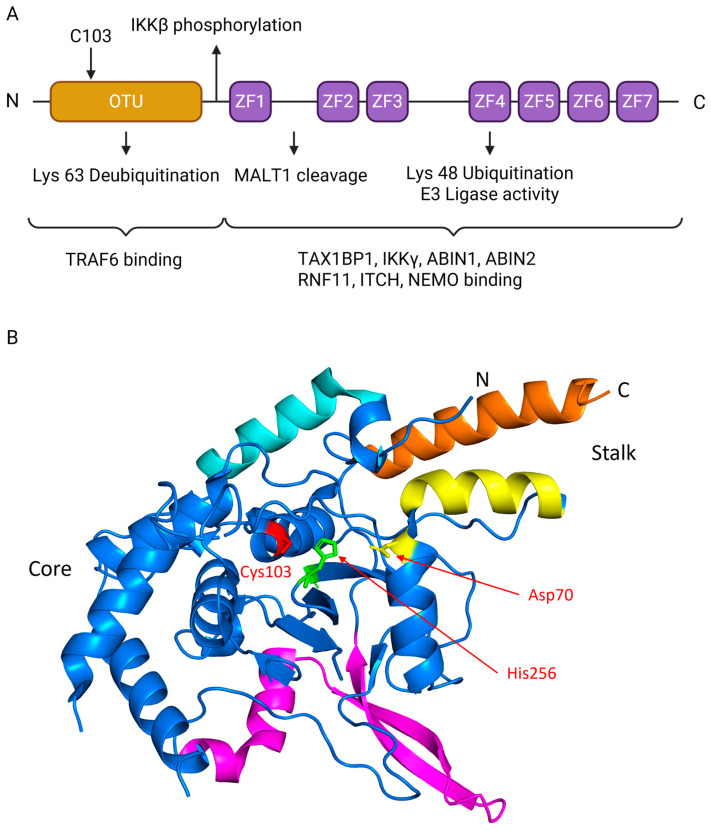
Structure of A20 protein. (**A**) Schematic representation of A20 domains, where the N-terminal OTU domain is colored in gold and the C-terminal zinc fingers (ZF1-ZF7) are colored in purple. The N-terminal OTU DUB domain contains catalytic Cys103 that removes K63-linked ubiquitin from substrates (e.g., TRAF6), damping NF-κB signaling. A linker near ZF1 carries an IKKβ site (classically Ser381) that tunes activity. The C-terminus contains seven zinc fingers (ZF1–ZF7); ZF4 provides E3-ligase activity that builds K48-linked chains for proteasomal turnover. MALT1 cleaves A20 between ZF1 and ZF2 (≈Arg439), transiently reducing inhibition. Partner recruitment maps to regions: N-term binds TRAF6, while ZF4–ZF7 engage TAX1BP1, IKKγ/NEMO, ABIN1/2, RNF11, and ITCH to assemble the ubiquitin-editing complex. (**B**) The crystal structure of the human A20 OTU domain. The image was generated using PyMOL (version3.1.6.1), based on coordinate data from the Protein Data Bank (PDB ID: 2VFJ). The structure is colored to highlight key regions identified in the original study: the core domain (blue), the α1 helix (cyan), the helical stalk formed by helix α2 (yellow) and orange (α11)), and the insertion domain (magenta). The essential catalytic triad residues—Cysteine 103 (red), Histidine 256 (green), and Aspartate 70 (yellow)—are shown as stick representations and explicitly labeled. Panel A created with BioRender.com (https://BioRender.com/t4hi4jt, accessed on 12 December 2025).

The modular domain structure of A20 is depicted in Figure 2A. The N-terminal OTU domain mediates deubiquitination of Lys63-linked chains, while the C-terminal zinc finger motifs, particularly ZF4, catalyze Lys48-linked ubiquitination for proteasomal degradation. These domains cooperate to enable A20’s “ubiquitin-editing” function. Post-translational regulation further fine-tunes activity, as exemplified by IKKβ-mediated phosphorylation or MALT1 cleavage. Moreover, recruitment of adaptor proteins through the C-terminal zinc finger region facilitates assembly of the A20 regulatory complex, ensuring substrate specificity and spatial precision [4,10,11].

To provide a structural foundation for understanding its function, we examined the crystal structure of the human A20 OTU domain, previously determined by Komander and Barford [12]. The structure, visualized from the Protein Data Bank (PDB ID: 2VFJ), reveals a complex fold characterized by a central core, a helical stalk, and a unique insertion region not commonly found in other OTU family members (Figure 2B). The active site is located within a conserved cleft and features a canonical catalytic triad essential for its deubiquitinase function. This triad is composed of Cysteine 103, which acts as the nucleophile, alongside Histidine 256 and Aspartate 70, which facilitate the catalytic reaction. This established structural framework of the OTU domain, complemented by the functions of the C-terminal zinc fingers, provides a basis for dissecting the multifaceted roles of A20 in ubiquitin editing and signal termination.

### 2.3. Functional Mechanisms in Cellular Signaling

These structural insights inform understanding of A20’s functional mechanisms in cellular signaling pathways. A20’s best-characterized role is negative regulation of NF-κB signaling. Following activation of tumor necrosis factor receptor 1 (TNF-R1), A20 removes activating Lys63-linked ubiquitin chains from the adaptor kinase receptor-interacting protein 1 (RIP1) and then deposits degradative Lys48-linked chains on the same molecule, targeting it for proteasomal destruction [4]. In Toll-like receptor (TLR) pathways, A20 performs a similar ubiquitin-editing step on the signaling adaptor TNF receptor-associated factor 6 (TRAF6), stripping its Lys63-linked chains to dampen downstream NF-κB activation [9].

This ubiquitin-editing mechanism is essential for terminating NF-κB signaling. A20 operates within a regulatory complex comprising adaptor proteins such as A20-binding inhibitor of NF-κB activation 1 (ABIN1) and Tax1-binding protein 1 (TAX1BP1), together with RING-type E3 ligases including RING finger protein 11 (RNF11) and Itchy E3 ubiquitin protein ligase (ITCH). These partners confer substrate specificity and spatiotemporal control [1,2,13].

**Figure 3:**
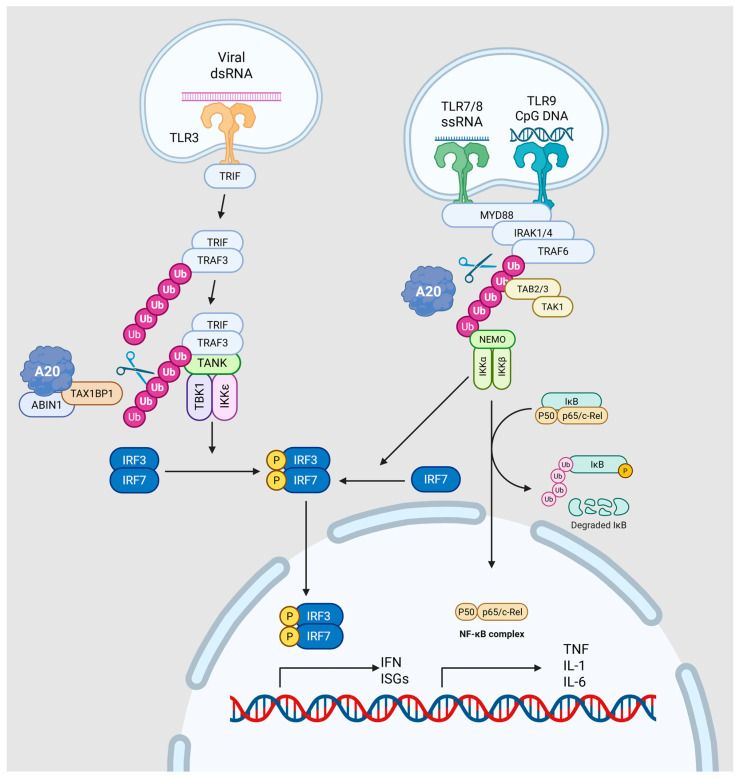
Schematic of A20 control of endosomal TLR signaling. Left, TLR3–TRIF axis: viral dsRNA activates endosomal TLR3, recruiting TRIF and then TRAF3, which scaffolds the TANK–TBK1/IKKε module. K63-linked ubiquitin platforms enable TBK1/IKKε to phosphorylate IRF3/IRF7, inducing type I IFNs and ISGs. The A20 regulatory complex (A20–TAX1BP1–ABIN1) edits ubiquitin chains on TRIF/TRAF3, removing K63 linkages to dampen TBK1/IKKε activation and IRF3/7 signaling. Right, TLR7/8/9–MyD88 axis: MyD88 triggers IRAK1/4 → TRAF6 → TAB2/3–TAK1, activating the NEMO–IKKα/β complex, which phosphorylates and degrades IκB to unleash NF-κB and drive TNF, IL-1, and IL-6 expression; MyD88 also promotes IRF7 activation. A20 restrains this branch by deubiquitinating TRAF6/NEMO and redirecting ubiquitin toward K48-linked turnover, thereby limiting both NF-κB and IRF7 outputs. Solid arrows indicate the signal transduction pathway. Purple and pink spheres represent ubiquitin chains. Yellow circles labeled ‘P’ denote phosphorylation events. The scissors icon indicates the deubiquitinating activity of A20. Figure created with BioRender.com (https://BioRender.com/j6ekhsu, accessed on 12 December 2025).

As illustrated in Figure 3, A20 regulates two major endosomal TLR pathways. In the TLR3–TRIF axis, the A20–TAX1BP1–ABIN1 complex removes K63-linked ubiquitin chains from TRIF and TRAF3, reducing TBK1 and IKKε activation and limiting IRF3/IRF7-mediated induction of IFNs and interferon-stimulated genes (ISGs). In the TLR7/8/9–MyD88 axis, A20 deubiquitinates TRAF6 and NEMO, promoting K48-linked ubiquitin-dependent degradation. This process suppresses IKK-driven IκB degradation and NF-κB transcriptional activity, while also dampening MyD88-dependent activation of IRF7.

### 2.4. Broader Biological Functions Beyond NF-κB

Recent studies show that A20’s influence extends far beyond NF-κB inhibition. Independently of NF-κB, A20 potently suppresses apoptotic and necroptotic cell death, preventing the release of damage-associated molecular patterns (DAMPs) that would otherwise amplify inflammation [5]. Through this cell-protective function, A20 also acts as a tumor suppressor. Loss-of-function mutations are frequent in various lymphomas and in colorectal carcinoma, underscoring its role in curbing aberrant survival and proliferation of malignant cells [6,7].

During viral infection, A20’s activities generate a complex interplay between host and pathogen. Many viruses exploit A20’s ability to dampen type I IFN production (e.g., TBK1/IKKε–IRF3 axis), creating a more permissive environment for replication and making A20 a proviral factor [1,2]. Conversely, by restraining excessive inflammatory cascades and lethal cytokine storms, A20 protects host tissues and improves survival, revealing a host-protective facet [8]. The outcome depends on the viral species, infected cell type, and stage of infection.

This review synthesizes the current knowledge regarding the dual and sometimes opposing roles of A20 during viral infections. We outline its proviral and antiviral functions across a range of viral pathogens and discuss how A20 modulates the balance of host–pathogen interactions that ultimately determines disease outcomes.

## 3. Proviral Effects of A20

### 3.1. Hepatitis C Virus

Hepatitis C virus (HCV), a (+) single-stranded RNA virus of the *Flaviviridae* family [14], exhibits high genetic diversity due to its error-prone replication. The envelope glycoproteins E1 and E2 mediate cell entry and serve as main targets for neutralizing antibodies. Although acute infection is often asymptomatic, a substantial proportion progresses to chronic disease, leading to hepatitis, cirrhosis, and hepatocellular carcinoma. Direct-acting antivirals (DAAs) have treatment outcomes, but the lack of a preventive vaccine highlights the importance of structural studies on the E1E2 complex [14,15].

During HCV infection, A20 acts as a proviral factor, supporting viral persistence through both direct enhancement of replication and suppression of host immune responses. HCV employs a mechanism whereby A20 transcription is induced by removing USF-1, a transcriptional suppressor at the A20 promoter, through proteasome-mediated degradation. Consistent with Figure 1, USF-1 acts at the ELIE to restrain promoter-proximal elongation via DSIF; its removal lifts this brake and rapidly activates A20 transcription. This induced A20 expression promotes the activity of the internal ribosome entry site (IRES) of HCV RNA, increasing translation efficiency of viral proteins [16].

Additionally, A20 facilitates immune evasion by suppressing host innate immunity. HCV antigen induces expression of A20 and ABIN1 in macrophages, which suppresses NF-κB signaling and consequently inhibits macrophage polarization toward the M1 type that is essential for mounting an effective inflammatory response [17]. Furthermore, A20 is overexpressed in myeloid dendritic cells (mDCs) from chronic HCV patients, where it inhibits mDC maturation and blocks antiviral cytokine (IL-12) production, contributing to establishment and maintenance of chronic infection [18]. This exploitation of A20’s regulatory functions demonstrates how HCV establishes persistent infection through simultaneous enhancement of replication and immunosuppression.

### 3.2. Avian Leukosis Virus Subgroup A

Avian leukosis virus subgroup A (ALV-A), a retrovirus of the family *Retroviridae*, genus *Alpharetrovirus*, with a (+) single-stranded RNA genome, replicates via reverse transcriptase [19]. Subgroups A–K are classified by Env variations, with subgroup A binding the avian Tva receptor to define host range. Infection induces tumors and immunosuppression, causing major economic losses in poultry. With no effective vaccines or treatments, control relies mainly on identifying and culling infected birds [19].

During ALV-A infection, A20 acts as a proviral factor, establishing positive feedback that enhances both viral replication and oncogenic potential. ALV-A directly promotes its own replication through a positive feedback mechanism involving A20. In chicken fibroblast DF-1 cells, ALV-A infection and A20 expression mutually promote one another, with viral challenge elevating A20 levels while A20 in turn suppresses TRAF6 ubiquitination, a key step in NF-κB activation [20]. This relationship is confirmed in vivo, where one-day-old chicks infected with ALV-A and engineered to overexpress A20 displayed aggravated weight loss, higher viremia, and blunted antibody responses, demonstrating that virus-induced A20 potentiates ALV-A pathogenicity.

Moreover, A20 enhances viral pathogenesis and oncogenesis through specific molecular mechanisms. In the DF-1 cell line study, the mutual promotion between A20 and ALV-A extends beyond replication to include oncogenic transformation. A20 not only inhibits TRAF6 ubiquitination but also promotes STAT3 phosphorylation. Phosphorylated STAT3 subsequently promotes expression of the proto-oncogene c-Myc, increasing the likelihood of tumorigenesis [20]. These mechanisms are further demonstrated in vivo, where chicken embryos inoculated with A20-overexpressing recombinant adenovirus and subsequently infected with ALV-A as one-day-old chicks showed the highest viremia and shedding rates compared to control groups [21]. These findings support that A20 overexpression promotes ALV-A replication while simultaneously enhancing its overall pathogenicity, including both viremia and oncogenic potential.

### 3.3. Bovine Viral Diarrhea Virus

Bovine viral diarrhea virus (BVDV), a (+) single-stranded RNA pestivirus of the *Flaviviridae* family [22], is a major ruminant pathogen causing reproductive, respiratory, and gastrointestinal diseases with substantial economic losses [23]. The envelope glycoprotein E2, a key neutralizing antigen, is central to vaccine design. Recent work shows that adjuvanted recombinant E2 subunit vaccines can induce antibody responses and protection, offering an alternative to traditional live-attenuated or inactivated vaccines [23].

During BVDV-1 infection, A20 acts as a proviral factor, facilitating viral persistence through suppression of host innate immunity. BVDV-1 employs A20 for immune evasion following a pattern comparable to other immunosuppressive viruses. In MDBK kidney cells, BVDV-1 infection elevates A20 expression, which subsequently suppresses phosphorylation of NF-κB p65 and reduces IL-8 expression [24,25]. Forced overexpression of A20 in MDBK cells suppresses NF-κB signaling and lowers transcription of downstream inflammatory mediators, including IL-8 [24]. During BVDV-1 infection, A20 is induced via the NF-κB pathway, positioning A20 as a feedback inhibitor that curtails excessive pathway activity [25].

These findings support a model in which A20 serves as a negative regulator of NF-κB–mediated inflammatory outputs in bovine epithelial cells, contributing to the immunosuppressive phenotype observed during BVDV-1 infection [24,25]. This experimental evidence demonstrates how the virus exploits A20’s regulatory functions to dampen host immune responses and establish a favorable environment for replication and persistence.

### 3.4. Sendai Virus

Sendai virus (SeV), an enveloped virus of the family *Paramyxoviridae* with a (–) single-stranded RNA genome [26], primarily causing respiratory disease but also showing neurotropism in young mice [27]. Its accessory C proteins regulate RNA synthesis and suppress macrophage activity, enhancing pathogenesis [28,29]. In the CNS of suckling mice, SeV persists by initially infecting ependymal cells, then spreading to neurons, where limited glycoprotein expression allows evasion of host immunity [30].

A20 acts as a proviral factor during SeV infection, facilitating immune evasion by suppressing innate immune signaling. As a ubiquitin-editing enzyme, A20 functions as a negative feedback regulator that prevents excessive activation of innate immune pathways, and SeV hijacks this regulatory mechanism to dampen antiviral defenses and create an environment favorable for viral persistence and spread.

Mechanistically, A20 accomplishes this suppression through multiple pathways. It directly binds the TLR3 adaptor TRIF [31] and simultaneously blocks RIG-I signaling by curtailing IRF3 activation [32]. This dual targeting of both TLR3 and RIG-I pathways represents a strategy whereby SeV exploits A20’s natural regulatory functions to dismantle the host’s antiviral response, turning the host’s own immune regulatory machinery against itself to establish and maintain infection.

### 3.5. Vesicular Stomatitis Virus

Vesicular stomatitis virus (VSV) is an enveloped, bullet-shaped, (–) single-stranded RNA virus in the family *Rhabdoviridae,* genus *Vesiculovirus* [33,34]. VSV initiates infection by attaching to the low-density lipoprotein receptor (LDLR) and entering the host cell through clathrin-mediated endocytosis [33,35]. Although VSV is a livestock pathogen causing vesicular stomatitis [33,34], its well-characterized biology and the development of reverse genetics systems have transformed it into a powerful platform for oncolytic virotherapy and as a vaccine vector, exemplified by the clinically approved Ebola vaccine [33,34].

**Figure 4:**
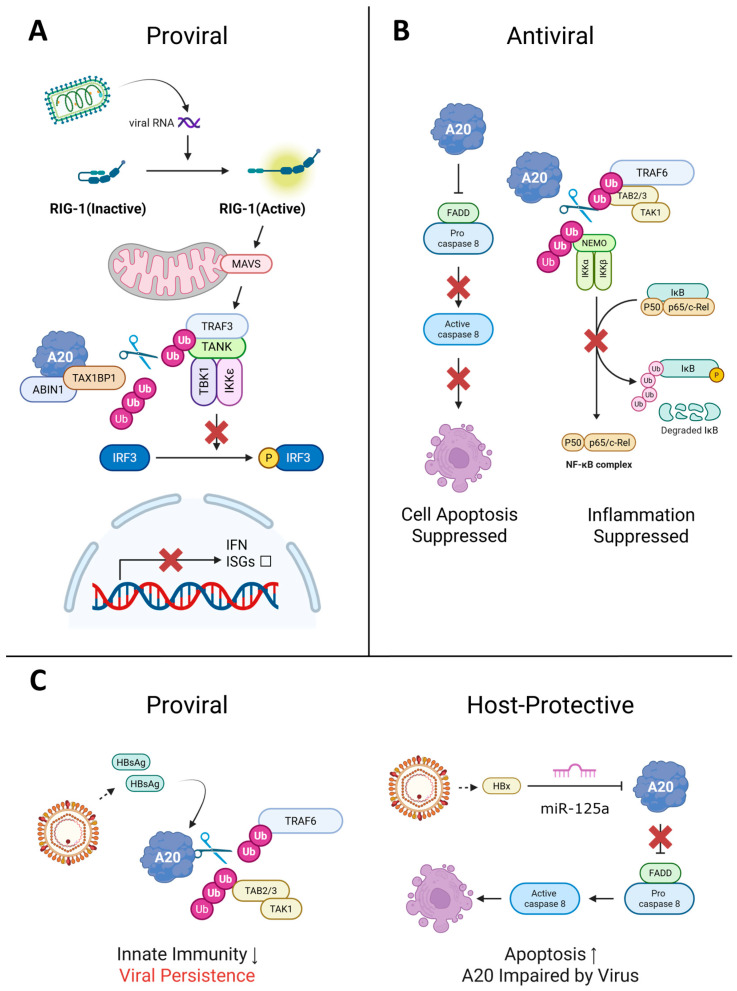
Roles of A20 in viral infection: proviral, antiviral, and host-protective functions. A20 (TNFAIP3) functions as either a proviral or antiviral factor depending on the virus, cell type, and infection stage. (**A**) Proviral: The A20–TAX1BP1–ABIN1 complex removes K63-linked ubiquitin chains from TRAF3 and TBK1/IKKε, blocking IRF3 phosphorylation and type I interferon (IFN) production. Viruses such as VSV and SeV exploit this mechanism to suppress antiviral immunity. (**B**) Antiviral: A20 prevents apoptosis by binding FADD and pro-caspase-8, blocking death-inducing signaling complex (DISC) assembly (left). Simultaneously, A20 deubiquitinates TRAF6 and NEMO to suppress NF-κB-driven inflammation (right), as observed in poliovirus and CVB3 infections. (**C**) Dual roles: A20 exhibits opposing functions depending on the cellular context, as exemplified by HBV. In monocytes (left), HBsAg-induced A20 disrupts TLR4 signaling by deubiquitinating TRAF6 and preventing TAK1–TAB2/3 complex formation, facilitating immune evasion and viral persistence. In hepatocytes (right), HBx upregulates miR-125a, which degrades A20 mRNA and enhances caspase-8-mediated apoptosis. TNFAIP3 polymorphisms impairing A20 function are associated with increased susceptibility to chronic HBV infection. Arrows indicate signal transduction. The red ‘X’ denotes a blocked pathway. Abbreviations: Ub, ubiquitin; scissors, deubiquitination. Figure created with BioRender.com (https://BioRender.com/83k7lck, accessed on 12 December 2025).

A20 acts as a proviral factor during VSV infection, creating a permissive environment for replication through suppression of host innate immunity [32]. VSV exploits A20-mediated dampening of type I IFN production, particularly along the TBK1/IKKε–IRF3 axis that is engaged downstream of RIG-I and depends on the E3 ligase Riplet for efficient signaling [36,37]. This targeting of the IFN signaling cascade helps VSV evade antiviral defenses and sustain infection (Figure 4A).

### 3.6. Human Coronavirus 229E

Human coronavirus 229E (HCoV-229E) is a (+) single-stranded RNA virus in the family *Coronaviridae*, genus *Alphacoronavirus* that causes mild, cold-like respiratory symptoms in humans [38,39]. Its spike glycoprotein binds the human aminopeptidase N (CD13) receptor [39,40], with receptor-binding domains shifting between closed and open states to enable attachment [38,40]. Extensive N-linked glycosylation shields the spike protein from immunity, and infection leads to marked downregulation of CD13 on host cells [39].

During HCoV-229E infection, A20 functions as a proviral factor by attenuating innate immune signaling and promoting viral replication. HCoV-229E directly promotes replication by employing A20 as part of a strategy to attenuate NF-κB signaling without fully activating it. This immunomodulation is achieved through multiple mechanisms that reduce protein levels of the IKK complex components (IKKβ, NEMO), induce incomplete degradation of IκBα, and strategically induce expression of A20 [41].

The importance of A20 in supporting replication is demonstrated by functional studies showing that knockdown of the A20 gene results in a 70% reduction in viral replication [41]. This decrease in viral output upon A20 depletion confirms that the virus actively induces A20 expression to suppress excessive antiviral responses, establishing A20 as a key proviral factor essential for HCoV-229E replication and persistence within infected cells.

### 3.7. Human Cytomegalovirus

Human cytomegalovirus (HCMV) is a double-stranded DNA virus with the largest genome (approx. 235–250 kb) among human herpesviruses [42,43]. Belongs to the family *Orthoherpesviridae,* genus *Cytomegalovirus*. HCMV is establishes lifelong latency with periodic reactivation [42]. While mostly asymptomatic in immunocompetent individuals, it can cause severe illness and death in the immunocompromised, such as organ transplant recipients and people with AIDS [42,43]. It is the most common infectious cause of congenital birth defects, leading to permanent disabilities like hearing loss, visual impairment, and neurological disorders in newborns [42,43]. To date, there is no licensed vaccine [43].

During HCMV infection, A20 functions as a proviral factor. A20 directly promotes viral replication through a temporal regulation pattern that reflects the virus’s replication strategy. HCMV infection reveals a unique biphasic pattern of A20 expression. A20 levels increase early in infection and subsequently decrease in later stages, representing viral manipulation of host regulatory mechanisms. The initial increase in A20 is mediated by the viral immediate early protein 1 (IE1), which directly activates A20 transcription through the NF-κB binding site in the A20 promoter, while the later decrease is attributed to epigenetic suppression by newly synthesized viral components [44].

The significance of this temporal regulation is demonstrated by experimental evidence showing that A20 knockdown significantly reduces viral replication, confirming A20’s role in supporting HCMV propagation [44]. HCMV utilizes the early upregulation of A20 to suppress excessive immune responses during initial infection phases, creating a cellular environment that facilitates replication. Subsequent downregulation of A20 may prevent prolonged immunosuppression that might compromise viral spread or persistence.

### 3.8. Human Respiratory Syncytial Virus

Human respiratory syncytial virus (HRSV) is a (–) single-stranded RNA virus in the family *Pneumoviridae*, genus *Orthopneumovirus*. HRSV is a major cause of bronchiolitis and pneumonia in infants and the elderly [45]. Its fusion (F) and attachment (G) proteins are key antibody targets, with the conserved F protein central to vaccine and monoclonal antibody design [45,46]. While antibodies such as palivizumab and nirsevimab are used for prevention, genomic studies reveal emerging epitope mutations, highlighting the need for continuous surveillance against resistant strains [46].

A20 acts as a proviral factor during HRSV infection by forming a deubiquitinase complex that suppresses innate immune signaling. HRSV infection of A549 epithelial cells leads to rapid accumulation of both A20 transcripts and protein, establishing an environment that favors viral replication [47]. The importance of A20 in suppressing antiviral responses is evident through functional studies. Genetic ablation or siRNA-mediated silencing of A20 restores a robust innate immune signature, characterized by upregulation of key cytokines including IL-6 and IFN-β [47].

Moreover, this mechanism requires formation of a complete deubiquitinase complex. Depletion of ABIN1 or TAX1BP1—partners that form the A20 deubiquitinase complex—produces identical restoration of cytokine responses [2,48]. Disruption of the A20 complex lowers viral titers, indicating that HRSV exploits A20-mediated suppression of immunity to promote replication while blunting host antiviral defenses.

### 3.9. Measles Virus

Measles virus (MeV), an enveloped (–) single-stranded RNA virus in the family *Paramyxoviridae*, genus *Morbillivirus* [49], uses hemagglutinin (H) and fusion (F) proteins to infect immune cells via SLAM (CD150), driving lymphotropism and immunosuppression (“immune amnesia”). Nectin-4 on epithelial cells enables respiratory transmission [49,50,51]. Beyond acute disease, MeV can persist in the brain, causing fatal subacute sclerosing panencephalitis (SSPE) [52]. Although highly antigenically stable and vaccine-preventable, global measles cases are resurging due to declining vaccination rates [49,51]

During MeV infection, A20 functions as a proviral factor, facilitating immune evasion through cell-type-specific suppression of host innate immunity. MeV exhibits cell-type-specific immunomodulatory effects, with monocytic cells showing A20 upregulation upon infection, while infected epithelial cells do not. This targeted upregulation of A20 in monocytes disrupts TLR4-mediated signaling by preventing formation of the TRAF6–TAK1–TAB2 complex, blocking downstream NF-κB activation and suppressing proinflammatory cytokine production [53].

The significance of this A20-mediated suppression is confirmed by experimental evidence. Silencing A20 using siRNA restores the LPS-induced signaling cascade, directly demonstrating A20’s role in MeV-mediated immunosuppression. In contrast, epithelial cells infected with MeV show no change in A20 expression or evidence of NF-κB pathway suppression, suggesting that the virus selectively manipulates innate immune responses in monocytes while sparing epithelial cells from such modulation [53]. This differential targeting creates localized niches that support viral persistence without compromising epithelial functions required for replication and spread.

## 4. Antiviral Effects of A20

### 4.1. Poliovirus

Poliovirus, a non-enveloped (+) single-stranded RNA enterovirus in the family *Picornaviridae,* genus *Enterovirus* [54,55], causes poliomyelitis via fecal–oral transmission and CNS infection. Its icosahedral capsid (VP1–VP4) binds the poliovirus receptor (Pvr), triggering conformational changes for cell entry [54,55]. Replication occurs in the cytoplasm, and global eradication efforts depend on inactivated (IPV) and live-attenuated oral (OPV) vaccines [54].

A20 acts as an antiviral factor during poliovirus infection, demonstrating inhibition of viral replication through mechanisms that highlight the host’s preservation of immune regulatory pathways even under severe viral suppression. The antiviral effect of A20 has been demonstrated in poliovirus infection models, where the virus creates a restrictive cellular environment by suppressing transcription of most host genes [56]. However, host cells selectively maintain transcription of key immune regulatory genes, including IκBα, A20, IL-6, and CCL2, many of which have NF-κB binding sites in their promoters, suggesting a response that prioritizes antiviral factors [56].

When A20 expression is reduced by siRNA, both viral RNA levels and viral yield increase, indicating that A20 constrains poliovirus replication [56]. These observations show that A20 is preserved during virus-mediated transcriptional shutdown to help control poliovirus infection.

### 4.2. Coxsackievirus B3

Coxsackievirus (CV), a non-enveloped, (+) single-stranded RNA virus in the family *Picornaviridae,* genus *Enterovirus*. Responsible for a wide spectrum of human diseases, from mild hand, foot, and mouth disease to severe conditions like myocarditis and meningitis [57,58]. The most studied serotype, coxsackievirus B3 (CVB3), infection by binding to the coxsackievirus and adenovirus receptor (CAR) and decay-accelerating factor (DAF) receptors [57]. The pathogenesis of CVB3-induced myocarditis progresses through distinct viral and autoimmune stages driven by innate and adaptive immune responses [57].

A20 plays host-protective roles during CVB3 infection, limiting immunopathology and preserving tissue homeostasis by restraining inflammatory signaling and apoptosis. Initially, CVB3 infection leads to decreased A20 expression in infected cells. Experimental manipulation reveals A20’s protective role. Artificial overexpression using lentivirus results in lower levels of inflammatory cytokines such as IL-6 and IL-18 compared to controls, while A20 knockdown conversely increases these cytokine levels [59].

This protective function is evident in the context of CVB3-induced viral myocarditis (VMC), where the molecular mechanisms underlying A20’s regulation are elucidated. CVB3 infection increases adenosine deaminase acting on RNA 1 (ADAR1) expression, which binds to Dicer and promotes maturation of pre-miR-1a-3p. This mature miRNA directly targets A20 mRNA to suppress its expression, leading to reduced A20 protein levels [59]. The consequences of this A20 downregulation are significant. Reduced A20 levels contribute to increased cell apoptosis and inflammation in myocardial tissue during CVB3 infection, while overexpression of A20 under identical infection conditions results in a reduced rate of cell apoptosis (Figure 4B).

This evidence demonstrates that A20 functions as a host defense mechanism that limits virus-induced tissue damage and maintains cellular homeostasis, with the virus targeting A20 for downregulation to facilitate pathogenesis.

## 5. Dual Effects of A20

### 5.1. Zika Virus

Zika Virus (ZIKV) is a (+) single-stranded RNA virus in the family *Flaviviridae*, genus *Orthoflavivirus*. Primarily transmitted by *Aedes aegypti* mosquitoes, infamous for causing severe congenital malformations such as congenital Zika syndrome (CZS), particularly microcephaly [60]. During its replication, ZIKV remodels the endoplasmic reticulum (ER) membrane to form viral replication organelles, a process in which it actively utilizes host proteins [60].

A20 plays a context-dependent regulatory role during ZIKV infection, with distinct observations in different cell types. In HeLa cells, ZIKV infection downregulates A20 protein expression despite elevated TNFAIP3 mRNA levels. The reduction in A20 protein occurs through post-transcriptional mechanisms rather than post-translational degradation, as neither autophagy inhibitors (Bafilomycin A1, chloroquine) nor the proteasome inhibitor MG132 restored A20 levels, and no MALT1-mediated cleavage was detected. ZIKV NS5, which localizes to the nucleus, and the capsid protein, which shows partial nuclear localization, were both identified as viral components responsible for decreasing A20 expression without directly interacting with A20 itself [61].

In parallel, ZIKV infection in HeLa cells led to sustained NF-κB activation and increased expression of prosurvival genes including BIRC3, BCL-2, and MCL-1. When A20 was overexpressed in ZIKV-infected HeLa cells, markers of cell death (CASP3-p17 and GSDME-p34) increased, suggesting that A20 restoration can promote apoptotic pathways in this context [61]. However, whether A20 protein is downregulated in ZIKV-infected macrophages remains to be directly demonstrated, as the underlying mechanisms were established primarily in HeLa and HEK293T cells.

A20’s function in viral infections is known to be highly context-dependent. While it generally acts as an anti-inflammatory regulator that limits NF-κB signaling and can restrict cell death in many settings, tissue-specific and expression-level-dependent variations have been reported where A20 may promote apoptosis [5]. This suggests that A20’s role in ZIKV pathogenesis may vary across different cell types and infection conditions [47].

### 5.2. Influenza A Virus

Influenza A virus (IAV) belongs to the family *Orthomyxoviridae* genus *Alphainfluenzavirus*, and has a genome of eight (−) single-stranded RNA segments, each packed as a ribonucleoprotein (RNP) complex [62,63]. Subtypes are determined by the antigenicity of two surface glycoproteins, hemagglutinin (HA) and neuraminidase (NA) (e.g., H1N1, H3N2), with 18 HA and 11 NA subtypes reported to date [62]. Due to its segmented genome, genetic reassortment (antigenic shift) can occur when different viruses co-infect the same cell, potentially creating new subtypes and causing pandemics [62,63]. The precise packaging of the eight genome segments into new virions is essential for viral replication and is tightly regulated by complex RNA secondary structures and inter-segment interactions [63].

A A20 displays both proviral and antiviral activities during IAV infection. Several studies in A549 cells have demonstrated that IAV infection induces a time-dependent increase in both A20 mRNA and protein. This upregulation is partly mediated by enhanced expression of the miR-29 family, particularly miR-29c, which stabilizes A20 mRNA through interaction with its 3′-UTR [64,65]. Additionally, the viral nonstructural protein NS1 promotes A20 protein accumulation without affecting transcript levels, particularly in highly pathogenic H5N1 and H7N1 strains [66]. Elevated A20 levels inhibit IRF3 phosphorylation and ISRE promoter activity, suppressing IFN-β and downstream ISGs such as IFITM3, ISG20, OAS1, and OASL, which facilitates increased production of viral M and NP proteins and elevated extracellular titers [66].

Beyond supporting replication, A20 also enhances IAV pathogenesis. In mice lacking A20, early infection stages are marked by elevated cytokine production (CCL2, IL-6) following TNF/poly I:C stimulation. However, in later stages, these mice exhibit reduced lung tissue damage and vascular leakage, correlating with decreased inflammatory cytokines and improved tolerance to infection. Restoration of CCL2 reverses this tolerance, confirming A20’s contribution to IAV-induced pathogenicity [67]. Furthermore, macrophage-specific A20 deficiency results in enhanced NF-κB and IRF3 activation in response to IAV infection, leading to elevated secretion of IL-6, TNF, and IFN-β, again supporting A20’s role in dampening early innate responses and allowing viral persistence [68].

A20 also displays antiviral properties by limiting host immunopathology. Its expression in airway epithelial cells helps suppress NF-κB–mediated inflammation. Chronic low-dose LPS exposure further enhances A20 expression and elevates PPAR-α and PPAR-γ levels, which in turn inhibit NF-κB and NLRP3 inflammasome activation. This mechanism protects against lung inflammation during IAV infection, maintaining tissue homeostasis and host viability [69].

### 5.3. Hepatitis B Virus

Belonging to the family *Hepadnaviridae*, hepatitis B virus (HBV) has a partially double-stranded DNA genome [70,71]. After infecting a host cell, HBV transports its genome into the nucleus and forms a stable covalently closed circular DNA (cccDNA). This cccDNA serves as the template for viral replication and is highly stable, which is a key factor in causing chronic infection and making treatment difficult [70,71]. HBV is a major cause of chronic hepatitis, cirrhosis, and hepatocellular carcinoma (HCC), making it a significant public health burden affecting approximately 300 million people globally [70,71].

A20 has both proviral and antiviral activities during HBV infection. As a proviral factor, HBV exploits A20 for immune evasion by suppressing host innate immunity. During HBV infection in THP-1 monocytes, the viral surface antigen HBsAg drives A20 expression, which subsequently deubiquitinates TRAF6, prevents TRAF6–TAB2 complex assembly, and halts TLR4 signaling (Figure 4C). This viral manipulation of A20 blocks LPS-driven cytokine production, as demonstrated by restoration of cytokine responses when A20 is suppressed by siRNA [72].

A20 also functions as an antiviral factor through multiple mechanisms. Genetic studies reveal that individuals with a specific polymorphism (TT>A variant) that impairs A20 function exhibit higher susceptibility to chronic HBV infection, indicating that fully functional A20 contributes to preventing viral persistence and facilitating viral clearance during early infection stages [73]. Additionally, A20 controls viral infection through regulation of apoptosis pathways. The HBV pathogenic factor HBx protein targets A20 for downregulation by inducing upregulation of miR-125a, which directly targets A20 mRNA and suppresses its expression, leading to caspase-8 activation and increased apoptosis (Figure 4C) [74]. This viral targeting suggests that A20 normally protects hepatocytes and prevents pathological damage, requiring the virus to overcome this protective function to induce disease.

The context-dependent nature of A20 is evident in its dynamic regulation during different phases of infection and varying clinical conditions. During the natural course of chronic HBV infection, A20 expression levels increase during the immune clearance phase when immune responses are active, due to TNF-α/NF-κB signaling pathway activation and representing a physiological feedback mechanism to restrain excessive immune responses [75,76]. This dual nature is apparent in conditions such as acute-on-chronic hepatic failure (ACHBLF) caused by HBV. In ACHBLF patients, A20 mRNA levels show positive correlations with liver injury indicators (total bilirubin, INR, MELD scores) and negative correlations with albumin and prothrombin time activity. Simultaneously, A20 levels correlate positively with IL-10 and negatively with IL-6, indicating suppression of inflammatory responses and induction of an immunosuppressive state. Among patients who died from ACHBLF, A20 mRNA levels were higher compared to survivors, exhibiting immune paralysis characterized by low IL-6 and high IL-10 levels [77].

### 5.4. Epstein–Barr Virus

Epstein–Barr virus (EBV), also known as human herpesvirus 4 (HHV-4), is a gamma-herpesvirus infecting over 90% of adults worldwide [78]. It targets B cells and epithelial cells to establish lifelong latency, classified into stages I–III by viral antigen expression. In latency I, Burkitt lymphoma cells express only EBNA1 to evade immunity, while proteins such as LMPs activate NF-κB and block apoptosis, driving oncogenesis. Through these mechanisms, EBV contributes to cancers including Burkitt lymphoma, Hodgkin lymphoma, and nasopharyngeal carcinoma [78,79,80].

A20 exhibits complex and opposing roles during EBV infection, functioning as both a proviral factor that supports viral persistence and an antiviral tumor suppressor. These context-dependent regulatory mechanisms demonstrate the intricate balance between viral exploitation and host defense.

In its proviral capacity, A20 plays a crucial role in prolonging infected cell survival and inhibiting apoptosis. Expression of the EBV oncoprotein latent membrane protein-1 (LMP1) in EBV-negative B-cell lines drives a pronounced increase in A20 at both transcript and protein levels [81]. LMP1-driven A20 expression effectively blocks p53-mediated apoptosis induction, with cells expressing A20 alone showing suppressed apoptosis to the same level as LMP1-expressing cells, independent of LMP1 itself [82]. The DUB domain at the N terminus of A20 directly binds to IRF7 and removes K63-linked ubiquitination, thereby facilitating immune evasion and contributing to both immune escape and cell survival. Studies demonstrate that A20’s deubiquitinase activity is essential for IRF7 inhibition, as DUB-deficient mutants completely lose the ability to suppress IRF7, while A20’s C-terminal domains are dispensable for this function [83].

However, A20 simultaneously functions as an antiviral factor by inhibiting virus-induced oncogenesis. A20 acts as a classical tumor suppressor gene that prevents malignant transformation by various oncogenic viruses, requiring viruses to evade or disable A20’s suppressive function to achieve tumorigenesis. This antiviral role is evidenced by frequent deletions or loss-of-function mutations in the A20 gene observed in various EBV-associated lymphomas, particularly AIDS-related lymphomas (ARL) [84,85]. The context-dependent nature of A20 is further demonstrated through its participation in dynamic regulatory mechanisms that control viral activity via negative feedback loops. Following EBV infection, A20 is upregulated as part of the host response and subsequently acts as a key component of sophisticated negative feedback regulation that suppresses upstream signaling to modulate immune response intensity. During EBV infection, LMP1 upregulates A20 expression via the NF-κB pathway, but A20 then physically binds to the LMP1 signaling complex and displaces signaling molecules such as TRAF1 and TRADD, thereby inhibiting LMP1-mediated NF-κB activation [86]. Simultaneously, A20 targets the LMP1-IRF7 pathway through its deubiquitinase activity, removing activating K63-linked ubiquitin chains from IRF7 to prevent excessive IFN responses while maintaining viral latency [83]. These dual negative feedback mechanisms illustrate how EBV exploits cellular regulatory networks to achieve a delicate balance between viral persistence and host cell survival.

### 5.5. Human T-Cell Leukemia Virus Type 1

Human T-cell Leukemia Virus type 1 (HTLV-1), which carries a (+) single-stranded RNA genome, was the first discovered human retrovirus. It primarily targets CD4+ T-cells, causing severe diseases such as adult T-cell leukemia/lymphoma (ATLL) and HTLV-1-associated myelopathy/tropical spastic paraparesis (HAM/TSP) [87,88]. Approximately 20 million people are infected worldwide, and while most remain asymptomatic carriers, about 5% develop disease [88]. ATLL is an aggressive malignancy with a very poor prognosis. Current treatment options include chemotherapy, targeted therapies, and allogeneic hematopoietic stem cell transplantation (allo-HSCT), while therapies using genetically modified stem cells are also under investigation [87].

A20 demonstrates paradoxical roles during HTLV-1 infection, serving simultaneously as a proviral factor essential for cell survival and as an antiviral tumor suppressor that must be overcome for oncogenic transformation. As a proviral factor, A20 is critical for prolonging cell survival and inhibiting apoptosis. Artificial depletion of A20 using shRNA in HTLV-1-positive cell lines results in significant growth inhibition, accompanied by activation and cleavage of caspase-8 and caspase-3/7. This protective function is mediated through A20’s formation of a stable physical complex with caspase-8 and FADD, an interaction facilitated by the zinc finger domain at the C-terminus of A20 [89,90].

Conversely, A20 also acts as a potent antiviral factor by inhibiting virus-induced oncogenesis. The key oncogenic protein Tax drives T-cell transformation through persistent NF-κB signaling, but for this oncogenic program to succeed, the virus must inactivate the A20 functional complex by exploiting the host molecule CADM1 [91]. This requirement for A20 inactivation demonstrates that A20 directly suppresses HTLV-1-mediated oncogenesis. The dual nature of A20 reveals the complex evolutionary pressure between HTLV-1’s need to maintain cell survival while simultaneously overcoming A20’s tumor suppressor activity to achieve malignant transformation.

## 6. Context-Dependent Effects of A20

### Human Immunodeficiency Virus

Human Immunodeficiency Virus (HIV), a (+) single-stranded RNA retrovirus, enters cells via its Env glycoprotein complex of gp120 and gp41. gp120 binds CD4, while gp41 mediates membrane fusion through a six-helical bundle, a major antiviral target [92,93]. Although once a fatal and untreatable disease, the advent of antiretroviral therapies has transformed HIV into a manageable chronic condition. Vaccine efforts now focus on stabilizing Env trimers. Early designs used gp120–gp41 disulfide bonds, whereas newer interdomain lock trimers within gp120 block CD4 binding yet retain broadly neutralizing antibody recognition, offering improved vaccine potential [93].

In HIV infection, A20 serves as a context-dependent regulator of host homeostasis and tissue integrity rather than a simple pro- or antiviral factor. A20 expression is significantly downregulated in PBMCs from viremic, treatment-naïve individuals compared to healthy controls, and its levels are partially restored with highly active antiretroviral therapy (HAART) [94]. This dysregulation directly impacts intestinal barrier function, as untreated viremia-associated type I IFN (specifically IFNα) suppresses A20 expression in intestinal epithelial cells, leading to cell death and compromised barrier integrity. Following antiretroviral therapy initiation, A20 restoration positively correlates with epithelial barrier markers claudin-4 (CLDN4) and tight junction protein 1 (TJP1) [95].

However, A20 also acts as an endogenous regulator of dendritic cell activation. While this prevents excessive inflammatory responses during natural infection, it can limit vaccine efficacy. In a murine model, immunization with A20-silenced murine dendritic cells (DCs), which become hyperactivated, elicited superior anti-HIV immune responses and induced robust mucosal immunity compared to control DCs [96]. These observations demonstrate that A20 is a critical host-protective factor in HIV infection. Its downregulation during viremia contributes to immunopathology and tissue damage, while its regulatory role in antigen-presenting cells represents a double-edged mechanism that protects against excessive inflammation but may constrain vaccine-induced immunity.

## 7. Discussion

The evidence reviewed here establishes A20 as a central, yet paradoxical, regulator of host–virus interactions [3,4]. Far from being a simple negative regulator of NF-κB [9], A20 operates as a multifaceted molecular switch that can either promote viral persistence or safeguard host tissues, depending on the viral species, infected cell type, and stage of infection [1,2]. This duality explains why A20 has been alternatively described as both a proviral factor and an antiviral defense mechanism [5,13].

A clear pattern emerges from comparative analysis (Table 1). For many RNA viruses including HCV [16,17,18], HRSV [47], MeV [53], and VSV [32], A20 is consistently hijacked to suppress innate immune signaling and facilitate replication, highlighting its proviral role. By contrast, in poliovirus and ZIKV infections, A20 exerts direct antiviral activity by sustaining essential immune responses or promoting apoptosis of infected cells, forcing these viruses to evolve countermeasures that suppress its expression [56,61]. Still, other pathogens, such as IAV [65,66,67,68,69], HBV [72,74,75,76,77], EBV [81,82,83,84,85,86], and HTLV-1 [89], exploit A20 during certain infection phases while being restrained by it in others. HIV represents a distinct case, where dysregulated A20 expression contributes to gut pathology and vaccine inefficacy, illustrating how its regulatory role extends beyond viral clearance to broader immune homeostasis [94,95,96].

These findings also carry important clinical and translational implications. The ability of A20 to fine-tune inflammation places it at the crossroads of viral pathogenesis and host survival [3,5,17]. Excessive immune activation often drives pathology into acute infections, whereas insufficient responses enable viral persistence and oncogenesis. Thus, therapeutic strategies aimed at modulating A20 require careful consideration. Pharmacological inhibition could restore antiviral immunity in chronic infections such as HBV [72,75,76,77] or HCV [16,18], yet risks triggering uncontrolled inflammation. Conversely, enhancing A20 activity might protect against immunopathology in diseases like influenza [69] or COVID-19 [8], but could simultaneously impair viral clearance [68].

Future research should prioritize several directions. Mapping A20’s interactome across different viral infections will be necessary to identify when its inhibition or activation is beneficial [1,2]. Genetic and epigenetic variation in A20 among human populations should be studied as potential predictors of viral susceptibility and clinical outcomes [97,98]. The development of targeted modulators—such as cell type-specific agonists or transient inhibitors—could enable precision immunotherapy that leverages A20’s regulatory capacity without triggering systemic toxicity [5,8,68].

In conclusion, A20 is best understood not as inherently proviral or antiviral, but as a dynamic immunoregulatory hub. Its actions reflect an evolutionary balance between host defense and tissue protection, continuously exploited and countered by viral pathogens. By dissecting how A20 operates in different infectious contexts, future studies may uncover therapeutic opportunities to both enhance antiviral immunity and minimize immunopathology, advancing host-directed antiviral strategies.

## Figures and Tables

**Table 1:** Effects of A20 on virus and host. A20 can act as a proviral, antiviral, or dual regulator depending on the virus and infection context. This table summarizes representative viruses in each category, their mechanisms of A20 modulation, and clinical/pathological implications. Symbols: → (leads to/results in); ↑ (increase/upregulation); ↓ (decrease/downregulation); *: (Highlight).

Effects	Virus	Mechanism of A20 Modulation	Clinical/Pathological Implications
Proviral *	HCV	USF-1 degradation → A20 ↑ → enhances IRES-mediated translation; suppresses NF-κB and DC maturation	Promotes chronic infection, immune evasion
	HRSV	A20+ABIN1/TAX1BP1 complex → suppresses IL-6, IFN- β	Maintains viral persistence in airway epithelium
	MeV	Monocytes: A20 ↑ → inhibits TRAF6 polyubiquitination → impairs formation/recruitment of the TAK1–TAB2–TRAF6 active complex → NF-κB suppressed	Cell-type–specific immune suppression
	VSV	A20 + ABIN1/TAX1BP1 complex antagonizes K63-linked polyubiquitination of TBK1/IKKε by disrupting the TRAF3–TBK1/IKKε module → IFN-I suppressed	Permissive environment for replication
	SeV	A20 binds TRIF and strongly blocks RIG-I/IRF3 activation	Suppression of the antiviral state → immune evasion
	HCoV-229E	Reduces IKKβ and NEMO; causes partial IκBα degradation; induces A20	A20 knockdown lowers infection/replication proxy (~70% ↓ by N protein)
	HCMV	Early: IE1 activates NF-κB–responsive A20 promoter → A20 ↑; Late/high MOI: de novo viral gene products epigenetically limit A20 transcription	A20 is required for efficient HCMV growth; biphasic control is suggested to support productive infection
	BVDV-1	A20 ↑ → NF-κB p65 phosphorylation ↓ → IL-8 ↓	Establishes immunosuppressive state
	ALV-A	Virus–A20 feedback; A20 inhibits K63-polyubiquitination of TRAF6 (→ TRAF6 protein ↑), leads to STAT3 phosphorylation ↑ → c-Myc ↑	In chickens, A20 up-regulation increases viremia and pathology; evidence supports contribution to oncogenesis
Antiviral	Poliovirus	Despite host shutoff, A20 transcription preserved; knockdown ↑ viral RNA/yield	A20 acts as an antiviral restriction factor
	CVB3	Virus induces ADAR1 → promotes miR-1a-3p maturation → A20 ↓; A20 overexpression → cytokines and apoptosis ↓	Protective against viral myocarditis
Dual	ZIKV	Virus downregulates A20 protein post-transcriptionally (TNFAIP3 mRNA ↑ but A20 protein ↓; ZIKV C/NS5 implicated) → inhibits apoptosis/pyroptosis	Supports cell survival and potential persistence
	IAV	Early: A20 ↑ → IRF3 inhibition, IFN-β suppressed (proviral); Late: NF-κB dampening reduces cytokine storm (host-protective)	Promotes replication in early phase; confers tissue-protective tolerance in later phase
	HBV	HBsAg → A20 ↑ (immune evasion); HBx/miR-125a post-transcriptional A20 ↓ → caspase-8 K63-Ub ↓, DISC ↑ → TRAIL-induced apoptosis ↑; TNFAIP3 TT>A variant increases susceptibility to chronic HBV	Linked to chronic infection and HCC progression
	EBV	LMP1 induces A20 via NF-κB; A20’s N-terminal DUB (OTU) domain binds IRF7 and reduces LMP1-driven K63-ubiquitination of IRF7 → IRF7 transactivation (IFN-I) suppressed; A20 also blocks p53-mediated apoptosis	Supports survival/persistence of infected cells (anti-apoptotic, IFN dampening); loss-of-function (deletions/mutations) of A20 in EBV-associated lymphomas sustains oncogenic NF-κB
	HTLV-1	Tax → A20 ↑ via NF-κB; A20 (C-terminal ZF) binds caspase-8 and FADD → blocks caspase-8 recruitment/activation → apoptosis suppressed; Tax requires CADM1/TSLC1 to inhibit IKKα → TAX1BP1 phosphorylation, disabling the A20–TAX1BP1 negative-feedback and sustaining NF-κB	Promotes survival of infected T cells; maintains constitutive NF-κB; contributes to leukemogenesis (ATL)
Context-dependent	HIV	Viremia: A20 ↓ (PBMCs/IECs) via IFN-I → epithelial barrier dysfunction (“leaky gut”); DCs: A20 restrains activation, blunting vaccine responses (mouse)	Drives mucosal immunopathology during viremia; DC A20 may limit vaccine efficacy

## Data Availability

Not applicable.
